# Analysis of Human Standing Balance by Largest Lyapunov Exponent

**DOI:** 10.1155/2015/158478

**Published:** 2015-03-18

**Authors:** Kun Liu, Hongrui Wang, Jinzhuang Xiao, Zahari Taha

**Affiliations:** ^1^School of Electrical Engineering, Yanshan University, Qinhuangdao 066004, China; ^2^College of Electronic and Information Engineering, Hebei University, Baoding 071000, China; ^3^Faculty of Manufacturing Engineering, University Malaysia Pahang, 26600 Pekan, Pahang, Malaysia

## Abstract

The purpose of this research is to analyse the relationship between nonlinear dynamic character and individuals' standing balance by the largest Lyapunov exponent, which is regarded as a metric for assessing standing balance. According to previous study, the largest Lyapunov exponent from centre of pressure time series could not well quantify the human balance ability. In this research, two improvements were made. Firstly, an external stimulus was applied to feet in the form of continuous horizontal sinusoidal motion by a moving platform. Secondly, a multiaccelerometer subsystem was adopted. Twenty healthy volunteers participated in this experiment. A new metric, coordinated largest Lyapunov exponent was proposed, which reflected the relationship of body segments by integrating multidimensional largest Lyapunov exponent values. By using this metric in actual standing performance under sinusoidal stimulus, an obvious relationship between the new metric and the actual balance ability was found in the majority of the subjects. These results show that the sinusoidal stimulus can make human balance characteristics more obvious, which is beneficial to assess balance, and balance is determined by the ability of coordinating all body segments.

## 1. Introduction

Balance is a complex physiological process involving the interaction of many body organs. Diseases from these organs can lead to weak equilibrium, which will affect people's daily lives, so an objective measurement for human balance maintenance dynamics is of great significance in disease diagnosis and rehabilitation treatment [1, 2]. The purpose of this research is to find an effective numerical indicator to measure human balance ability and to analyse the relationship between human balance ability and dynamic characteristics.

Traditional methods for assessing balance ability [3, 4], which depend on centre of pressure (COP) trajectory, are considered as a descriptive way of characterizing body movement patterns. These approaches are not sensitive enough to investigate human systemic dynamics [5]. Therefore, some nonlinear parameters based on the concept of chaos have been proposed to detect the important hidden dynamic properties of physiological signals [6–8]. In the field of human motion mechanism, [9, 10] confirm that human standing derives from chaotic dynamics by establishing a simulation model. Their results verified that the new analysis method by using chaos system parameters had a relationship with human balance.

The largest Lyapunov exponent (LLE) is a typical nonlinear parameter to quantify the chaotic behaviour of postural sway. In [11], LLE values, which were evaluated from COP time series, were positive and greater than zero. They claimed that the postural control system derives from a process exhibiting chaotic dynamics. A similar result was also found by Ladislao and Fioretti [12], who investigated the effect of different visual conditions on the postural steadiness time series of normal subjects along the anterioposterior (AP) direction using traditional linear posturographic measures and nonlinear dynamical system quantifiers. Pascolo et al. used LLE to distinguish healthy controls from Parkinson's disease patients [13]. They claimed that human postural control system is indeed chaotic and they also found low dimensional attractors for sway dynamics in both groups. They calculated positive LLE values for both healthy subjects and Parkinson's disease patients, but these values could not clearly discriminate healthy subjects from Parkinson's disease patients.

In the above studies, subjects stand on static support surfaces. Because of body's endogenous sway, the related biological signal of human movement may suffer from interference to a certain extent, or even be submerged in noise. It is not only unconducive to analyze the dynamic processes of the human body, but also it directly affects the numerical accuracy of LLE [14, 15]. Many researchers have tried an external force stimulus to improve the signal to noise ratio of human dynamic information. In [16], a random translational stimulus was applied to the feet by a movable support surface. However, the random sudden motion causes the body to remain in a stressed state for a long time. Because of the patients' physiological and psychological factors, the method may not be conducive to obtain objective balance adjustment data in clinical practice. In this work, attempts were made to use a sinusoidal AP motion in order to generate a periodic external disturbance to the plantar. If the amplitude and frequency of the motion platform were suitably selected, the phenomenon found in the experiment showed that the subjects' COP periodically swayed to track the moving platform. And the psychological stress of subjects could be reduced by the sinusoidal stimulus, by contrast with the sudden movement stimulus.

In addition, the current analysis methods for human standing balance are mainly based on LLE of one-dimensional COP time series, with a large amount of human dynamic information being lost. For complex systems, multidimensional time series contain more detailed dynamic characterization than one-dimensional time series [17–19]. The conclusion in [13], which claimed that the nonlinear dynamic system parameters cannot accurately distinguish between Parkinson's disease patients and normal controls, may be the result of a lack of dynamic data in sufficiently high dimension. In our experiment, a multiaccelerometer subsystem was utilized and captured multidimensional acceleration time series during the balance adjustment process.

In this paper, we propose that the standing balancing ability can be assessed by a metric, which can reflect the overall coordination between multisegment movements, when a sinusoidal external perturbation is applied to the plantar. Two improved designs are performed in the experiment. (1) A continuous sinusoidal moving platform is applied to generate external disturbance to the plantar, which makes the movement characteristics more obvious. (2) A multiaccelerometer system is attached on different body parts in order to obtain multidimensional acceleration time series. Twenty healthy students are divided into three groups, in accordance with their performance during the experiment, and then the chaotic parameters of the experimental data are calculated. By statistical analysis, the results indicate three aspects: firstly, the sinusoidal stimulus makes the movement characteristics more obvious, which is good for assessing balance. Secondly, regarding whether the sinusoidal stimulus exists or not, the LLE values from one-dimensional time series seem unable to distinguish individuals effectively. Thirdly, the balance ability is associated with the ability to coordinate all body segments in some extent.

## 2. Methodology

### 2.1. Subjects

Twenty healthy student volunteers participated in the experiment (10 females: f1~f10; 10 males: m1~m10. Numbers are random). Mean age is 25.7 ± 3.1 years; mean height is 169.6 ± 6.3 cm; mean weight is 61.6 ± 8.9 kg. All subjects reported having no muscle or neurological movement disorders history, and they identified themselves as healthy with the ability to stand comfortably for 20 min [8, 20].

### 2.2. Devices

#### 2.2.1. Motion Platform

The motion platform was controlled by a programmable motion controller (Canada Quanser Q8 motion control board), which provides AP sinusoidal external disturbance to the plantar of subjects.

#### 2.2.2. Sensors System

**Figure 1:**
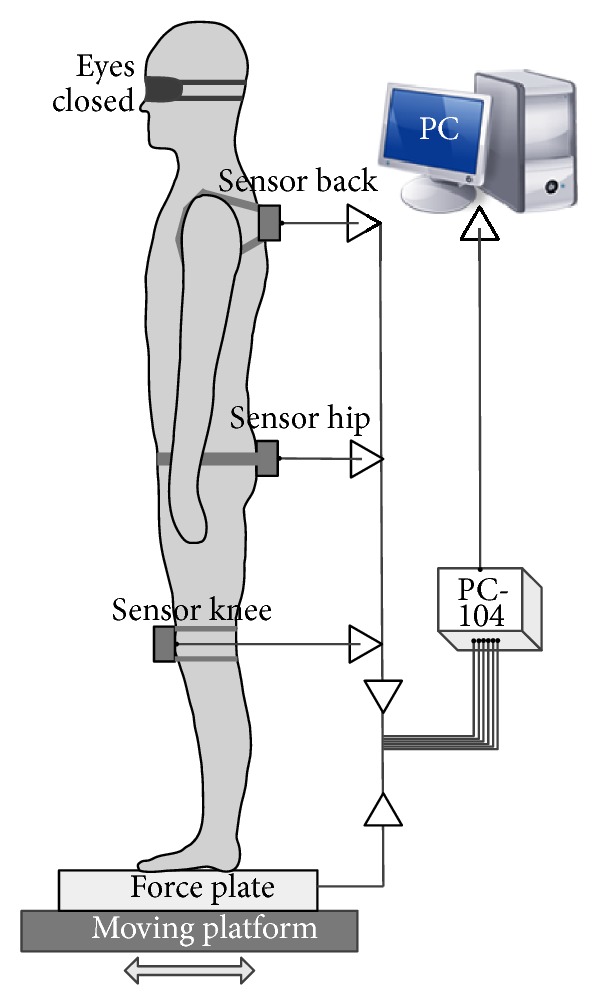
The posture of the subject and testing system. Accelerometers are attached to the back, hip, and knee. Subjects stand on the motion platform with upper arms along their respective sides. Two feet are apart at the same width as their shoulder-width. Eyes are closed. PC-104 collects and saves the real-time sway data of subjects. Motion platform generates AP sinusoidal external disturbance to plantar. COP data are obtained by the force plate.

*(1) Accelerometer*. MMA7361L (Freescale Semiconductor), which is fixed on the back, hip, and knee (Figure 1), is used to collect the subject's dynamic information. The sampling frequency is 100 Hz. The signals from 3 accelerometers represent the dynamic information of different body segments, which are lower segment (legs), middle segment (thighs), and upper segment (trunk, arms, and head) [20].

*(2) Force Plate*. An OPT400600 (AMTI) is used to obtain the COP position time series, whose measurement accuracy is typically ±0.1% of the applied load. Its base is fixed on the movable platform, and subject stands on the force plate during experiment.

*(3) Data Storage Module*. The PC/104 CPU Module (Em104P-i2904, ARBOR technology), which has a 6-channel 16-bit precision differential A/D converter, can save real-time sway data of the subject in a  .txt file onto the flash disc.

### 2.3. Experiment Method

After subjects filled in their basic personal information (height, body segment length, and weight), the accelerometers were attached to the predetermined position of body. The subjects stood on the motion platform with their upper arms along their respective sides, with their feet apart at the same width as their shoulder-width, as shown in Figure 1. In addition, some requirements were to be complied with: the subjects had to close their eyes to minimize any visual effects, try to maintain their best standing upright posture, without swinging their arms or moving their feet, and maintain their balance depending only on the major joints of their bodies.

Each subject was exposed to seven trials, and the duration of each was about 120 s, during which the platform underwent sinusoidal motion in the AP direction. The seven frequencies used were fixed at 0, 0.2, 0.4, 0.6, 0.8, 1.0, and 1.2 Hz. For each frequency, the amplitude used was fixed at a single peak of 25 mm. At the beginning of the first 20 s, subjects changed their sway rhythm to adapt to the sinusoidal motion. If the subjects experienced no discomfort, the data collection would last for 100 s; meanwhile, the actual performance of each subject was recorded.

### 2.4. Data Filtering

**Figure 2:**
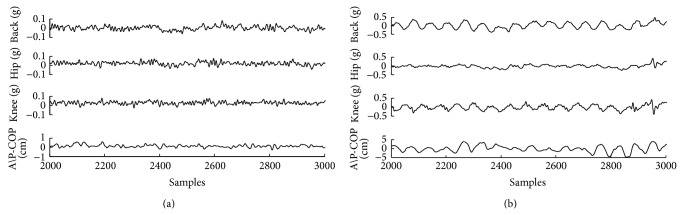
The raw data of a subject: (a) stimulus is in the 0 Hz, (b) stimulus is in the 0.8 Hz. The dynamical data is from a typical subject and (a) shows the data when the subject is standing under stimulus of 0 Hz frequency; (b) shows the data when the subject is standing under stimulus of 0.8 Hz frequency.

In order to analyse the dynamic character, linear filter processing is necessary to reduce the noise in raw data. In this work, a low pass of 2-order digital filter with 5 Hz cutoff frequency is applied, which is the same as in [11]. In the software environment of MATLAB 2010b, the raw data of body movements (the acceleration and the COP time series) were obtained. The data of a typical subject is shown in Figure 2.

Figure 2(a) shows the data when a subject is standing under a stimulus of 0 Hz frequency, and Figure 2(b) shows the data when the subject is standing under a stimulus of 0.8 Hz frequency. The acceleration time series of back, hip, and knee comes from the accelerometers. The COP time series is the component along AP direction.

### 2.5. State Space Reconstruction

It is necessary to reconstruct the state space of the dynamical process for calculating the nonlinear parameter by embedding time lag copies of the time series [21], which is [*x*(*t*), *x*(*t* + *τ*), *x*(*t* + 2*τ*),…, *x*(*t* + (*m* − 1)*τ*)]. The *τ* and *m* represent the embedding time lag and embedding dimension, respectively. The reconstructed attractor from the dynamical data must preserve the invariant characteristics of the original unknown attractor. Different from an infinite noise-free data set, the data set in experiment is finite and noisy; therefore, the choice of the delay time is important in the reconstruction of the attractor from the time series. Also, the optimal *τ* and *m*, which are unique to one dynamical system, are important to the LLE results.

C-C method [22], which seeks either time lag *τ* or time lag window *τ*_*w*_ by using the correlation integral, is adopted to reconstruct the state space of the dynamical system. C-C method is a combined algorithm for *m* and *τ*, because the *τ*_*w*_ is unique to one time series, and *m* and *τ* conform to the equation *τ*_*w*_ = (*m* − 1)*τ*. The accurate description of this method is in [22], while here we only pay attention to the results. By calculating the time series data of all subjects, reasonable reconstructing parameters set can be obtained (the average time lag *τ* = 30 ± 5.69; the average embedding dimension *m* = 4 ± 0.71). Since the optimal *τ* and *m* are unique to a dynamical system, the corresponding optimal *τ* and *m* should be applied to calculate the LLE for single time series.

### 2.6. Determinism Test and Stationarity Test

In real-life systems, sources of irregular behavior are perhaps from the ever-present noise. Therefore, after reconstructing the state space of human standing systems, determinism test and stationarity test are necessary to prove whether the studied systems have the typical properties of dynamical system. Assuming that these two tests are positive, one could then proceed to quantify the dynamics [23–25].

**Figure 3:**
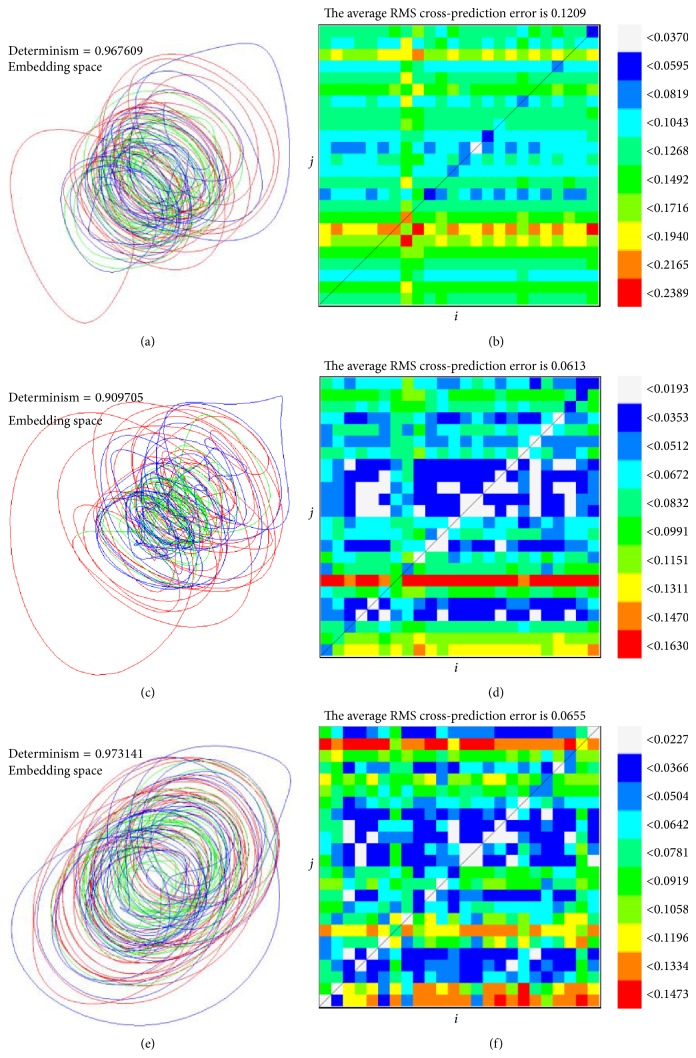
The results of determinism test and stationarity test for the acceleration time series of a typical subject. (a) and (b) are the results of back, (c) and (d) are the results of hip, and (e) and (f) are the results of knee. (a), (c), and (e) are the embedding space and (b), (d), and (f) are the average cross-prediction error for all the possible combinations of *i* and *j*. The average values of all *δ*_*ij*_ are 0.1209, 0.0613, and 0.0655 (for the acceleration time series of back, hip, and knee, resp.).

The determinism test, which was proposed by Kaplan and Glass [26], enables us to measure average directional vectors in the coarse grained embedding space. The embedding space should be coarse grained into equally sized boxes. Each pass *l* of the trajectory through the *k*th box is approximated to a unit vector *e*_*l*_, whose direction is determined by both space points where the pass *l* enters and leaves the box. Therefore, the average directional vector *V*_*k*_ of the *k*th box is

(1) $$\begin{array}{c} V_{k}=\frac{1}{R}\overset{R}{\underset{p=1}{\sum }}e_{l}, \end{array}$$

where *R* is the total of all passes in box *k*. If the time series originates from a deterministic system and the coarse grained partitioning is fine enough, the vector *e*_*l*_ inside one box may nearly not cross, and each crossing decreases the size of the average vector *V*_*k*_. Hence, for a deterministic system, the average length of all directional vectors will be 1, while for a random system it decreases to 0. In this section, the acceleration time series of a typical subject is evaluated by determinism test. The determinism factors are 0.968 (acceleration time series of back, *τ* = 28 and *m* = 4), 0.909 (acceleration time series of hip, *τ* = 31 and *m* = 4), and 0.973 (acceleration time series of knee, *τ* = 27 and *m* = 5) and the corresponding embedding spaces are shown in Figures 3(a), 3(c), and 3(e), which clearly confirms the deterministic nature of human balance system.

In order to verify if the studied sway is from a stationarity process, stationarity test, which is proposed by Schreiber [27], is evaluated for each data set by the recurrence plot analysis. In this method, the time series is divided into *h* nonoverlapping segments and *h* ^2^ possible combinations to calculate the statistics (*h* = 25). By calculating the average cross-prediction error (*δ*_*ij*_) for possible combinations of segments *i* and *j*, those dynamical changes in time series are shown obviously. If *δ*_*ij*_ is not significantly larger than the average value for any combination of *i* and *j*, it indicates that the time series sources from stationary system. In this section, the acceleration time series of a typical subject is evaluated by stationarity test. The average cross-prediction errors for all possible combinations of *i* and *j* are in Figures 3(b), 3(d), and 3(f). The average values of all *δ*_*ij*_ are 0.1209, 0.0613, and 0.0655 (for the acceleration time series of back, hip, and knee, resp.). Since each maximal cross-prediction error is not significantly larger than the average, the studied time series are clearly stationary.

### 2.7. Largest Lyapunov Exponent

For a dynamical system, its attractor trajectory contains the main dynamical characteristics, whose sensitivity to the initial condition represents deterministic chaotic characteristics [13]. The sensitivity to initial condition is quantified by LLE. If LLE is positive, the nonlinear deterministic system is chaotic. The greater the LLE value is, the more divergent the attractor is [28, 29]. The LLE of attractor is calculated by the algorithm in [30]. The initial distance between the *j*th point *x*_*j*_ and its nearest neighbor $x_{\hat{j}}$ is defined as

(2) $$\begin{array}{c} d_{j}\left(0\right)=\min\left\|x_{j}-x_{\hat{j}}\right\|,\;\left|j-\hat{j}\right|>\text{mean}\;\;\text{period}, \end{array}$$

for each point *x*_*j*_ in the phase space, the distance after the evolution of *i*th steps is defined as *d*_*j*_(*i*). Suppose that the *j*th point *x*_*j*_ and nearest neighbor $x_{\hat{j}}$ diverge at a rate given by *γ*, we have *d*_*j*_(*i*) = *d*_*j*_(0) × *e* ^*γ*(*i*·Δ*t*)^. By taking the logarithm to both sides, we obtain ln⁡*d*_*j*_(*i*) = ln⁡*d*_*j*_(0) × *γ*(*i* · Δ*t*). The LLE value is defined as a divergence curve 〈ln⁡*d*_*j*_(*i*)〉 versus  *i* · Δ*t*, which can be fitted by least squares

(3) $$\begin{array}{c} y\left(i\right)=\frac{1}{\Delta t}\langle \ln d_{j}\left(i\right)\rangle , \end{array}$$

where, 〈·〉 denotes the average over all values of *j*.

In order to investigate the coordination ability of the series structure system based on LLE, a simple metric, named coordinated LLE (CLLE), is presented, and defined by

(4) CLLE=LLEback−LLEhip2+LLEback−LLEknee2  +LLEknee−LLEhip21/2,

where LLE_back_, LLE_hip_, and LLE_knee_ are the LLE values for back, hip, and knee acceleration time series, respectively. The new metric is proposed to represent the difference of the chaotic dynamic of all body segments. And the chaotic dynamic of all body segments is integrated to compensate the defect of LLE from one-dimensional time series.

### 2.8. Analysis of Variance

A one-way analysis of variance (ANOVA) is performed by SPSS 19.0 (SPSS, Inc., Chicago, IL) to determine if there is statistical significance between different groups for assessing balance. The level of significant difference is 0.05. In hypothesis testing, the significance level is a criterion to reject the null hypothesis. The lower the significance level is, the more significant the data subset must diverge from the null hypothesis will be. If the *P* value of certain subset is less than the significance level (0.05), it concludes that data subset attains statistical significance compared with different other data.

## 3. Results

### 3.1. Performance Classing

During the experiment, all the 20 subjects were able to maintain balance in band of 0~0.8 Hz, so 0.8 Hz can be considered as a conservative upper limit of stimulus amplitude for all subjects. When the frequency was over 0.8 Hz, some participants would occasionally step or substantially sway, and when the frequency was raised to 1.2 Hz, five subjects were unable to complete the experiment. Therefore this indicates that the five subjects who could not withstand the frequency 1.2 Hz have poor balance control ability. Based on the different performance of subjects with 1.2 Hz stimulus, the subjects were divided into excellent, stable, and unstable groups:

 excellent group: f1, f4, m1, m3, m5, m7;
 stable group: m4, m6, m8, m9, m10, f2, f7, f9, f10;
 unstable group: f3, f5, f6, f8, m2,

where “excellent” means that their feet did not leave the support surface and their body swayed in a small amplitude without lifting the heels; “stable” means that their bodies swayed slightly more widely, with occasional lifting of foot or stepping; “unstable” means that their bodies swayed in a larger amplitude, always stepping or falling, and the subjects were unable to complete 100 s test.

When under lower intensity stimulus condition, the special dynamic characteristics of different individuals are also hidden in the motion. The more intense the perturbation is, the more obvious the dynamic characteristics of the human body will be. On one hand, the perturbation should not cause the body to step or fall from the support surface, which might harm the subject. On the other hand, this stimulus should be intense enough to effectively improve the characteristics of the body's movement. Therefore, the human signal of 0.8 Hz stimulus is the most suitable for the passive standing balance analysis. In addition, 0 Hz perturbation condition, which is regarded as a quiet standing condition, is the control condition.

### 3.2. LLE Results

The human motion data under the stimulus frequencies of 0 Hz and 0.8 Hz were calculated to measure the balance ability, while the standing performances under the stimulus frequency of 1.2 Hz are used to verify the metric. By using (3) and (4), LLE and CLLE values were solved from the human motion time series of the stimulus under 0 Hz and 0.8 Hz, and the results are listed in Tables 1 and 2. The columns LLE_back_, LLE_hip_, and LLE_knee_ are the LLE for time series from the accelerometers on back, hip, and knee, respectively. Column LLE_COP_ is the LLE time series of COP trajectory component in AP. The CLLE values are in the last column of Tables 1 and 2.

### 3.3. Statistical Results

**Figure 4:**
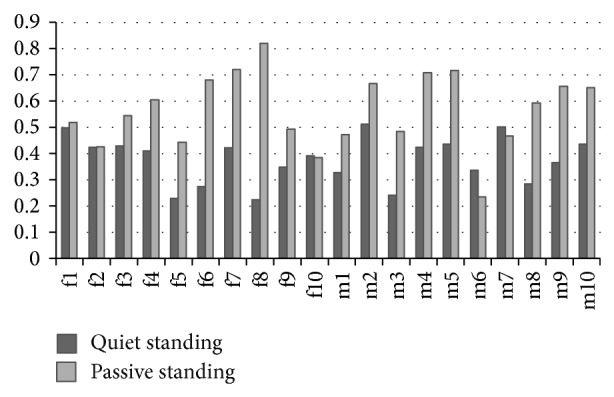
Comparison of quiet standing LLE_COP_ and passive standing LLE_COP_. The vertical axis represents the value LLE_COP_; the horizontal axis represents the sample number of subjects. Dark bar shows LLE_COP_ values in Table 1 (at 0 Hz platform motion, static sway data of the body without stimulus), while light bar shows LLE_COP_ values in Table 2 (the data of passive sway body under 0.8 Hz stimulus).

The quiet standing LLE_COP_ (Table 1) and passive standing LLE_COP_ (Table 2) are compared, which are shown in Figure 4. The vertical axis represents the LLE_COP_ value, and the horizontal axis represents the sample number of the subjects. The dark bar is LLE_COP_ value in Table 1 (quiet standing data, with the body under 0 Hz stimulus), and the light bar is LLE_COP_ value in Table 2 (passive standing data, with the body under 0.8 Hz stimulus).

**Figure 5:**
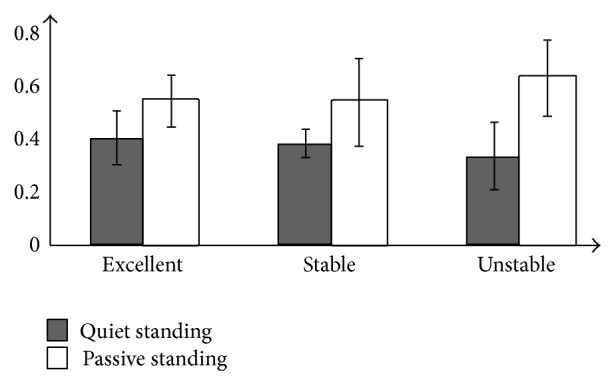
The average LLE_COP_ values of groups. The average LLE_COP_ values of excellent group, stable group, and unstable group are compared. Dark bar represents the quiet standing conditions, and the white bar represents the passive conditions.

In Figure 5, the LLE_COP_ mean values of the excellent, stable, and unstable groups are compared. The dark bar represents quiet standing condition, and the white bar represents passive standing condition.

**Figure 6:**
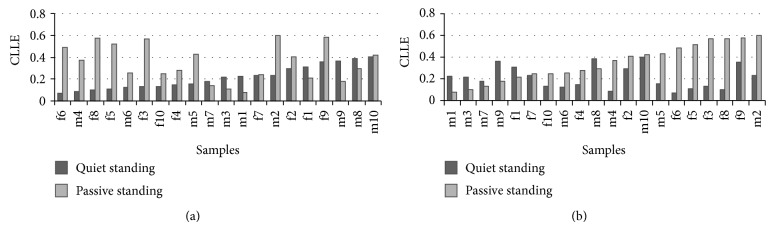
The CLLE values of all samples. The CLLE values of each sample are listed. (a) The sample number is in ascending order of quiet standing CLLE; (b) the sample number is in ascending order of passive standing CLLE.

The CLLE values of different samples are in Figure 6. (a) shows the sample number in ascending order of quiet standing CLLE, while (b) shows the sample number in ascending order of passive standing CLLE.

**Figure 7:**
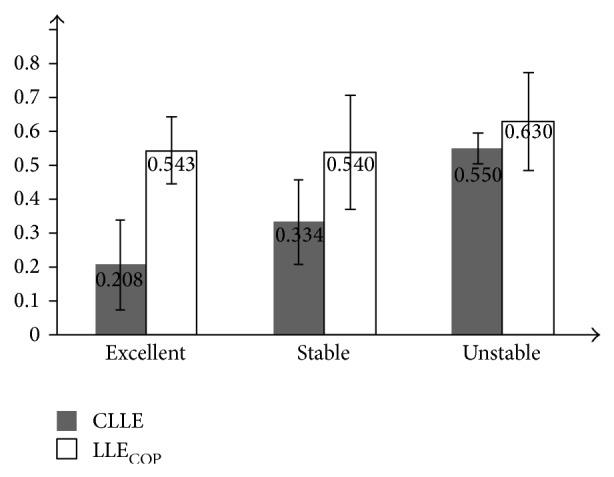
Group mean values of CLLE and LLE_cop_ from passive standing condition. The different metric means of each group are compared. Dark bar represents the passive standing CLLE mean value of groups; the white bar represents the passive standing LLE_COP_ mean value of groups.

In Figure 7, the CLLE and LLE_COP_ mean values of the three groups are compared. The dark bar represents the mean passive standing CLLE value, and the white bar represents the mean dynamic LLE_COP_ value.

In Table 3, the average quiet standing LLE values for the acceleration data are shown. The LLE average values for the acceleration data of body segment of excellent, stable, and unstable group are compared. The column represents the LLE values of different body segment, and the row represents different groups. The *P* values are in the last row of Table 3.

## 4. Discussion

In order to investigate the relationship between LLE and the standing balance ability, two measures were adopted. Chaotic parameters of the data set for 20 healthy students' are calculated. In discussion, these results will be further analysed.

*(1) What Role Does the Sinusoidal Stimulus Play for the Chaotic Characteristics?* The COP of quiet standing posture is mixed with white noise. Due to the low signal to noise ratio, dynamical characteristics are not measured directly. When a sinusoidal stimulus is applied with a reasonable amplitude and frequency, body sway changes speed as needed until its frequency matches the platform frequency. The phenomenon in this experiment shows that subjects can withstand the intensity of physiological and psychological pressure.

A number of findings have been reported by using a platform perturbation stimulus to human body in experiment. Schilling and Robinson [31] used an air bearing platform which was in sinusoidal AP motion in order to generate a periodic stimulus to postural control system, and then a mathematical model was established. Acharya et al., in [16], used platform perturbation whose acceleration was gradually increased from 1 m/s^2^to 5 m/s^2^to study the response of ten healthy subjects. The results showed that higher acceleration of the platform results in a lower LLE value, and lower acceleration of the platform results in a higher value. van der Kooij and de Vlugt [32] used pseudorandom translations of a platform in the ML direction with platform frequencies in a certain range. By spectral analysis, the COP and ankle torque responses were decomposed into periodic and remnant (stochastic) components. The results supported that balance control is based on a continuous feedback mechanism where observed variations in the responses are due to noise associated with state estimation errors.

In this study, LLE is applied as a metric to analyse human balance. The LLE_COP_, which is based on the AP component of COP time series, has been discussed as a data analysis indicator by many researchers [8, 12, 14]. In Figure 4, LLE_COP_ values in quiet standing and passive standing condition do not show the special relationship to different performances of the subjects. In Figure 5, the excellent and stable groups have approximately quiet standing LLE_COP_ mean values, while quiet standing LLE_COP_ mean value of the unstable group is lower than that of the other two groups, and the passive standing LLE_COP_ mean value of unstable group is higher than the other two groups. Compared with quiet standing LLE mean value, the difference of passive standing LLE mean value in the unstable group and other groups is obvious. These results imply that sinusoidal stimulus from the motion platform plays a role in increasing the dynamical characteristic of body movement. The difference of the LLE mean values becomes bigger from quiet standing to passive standing condition.

The LLE of one-dimensional time series seems unable to distinguish individuals into three groups effectively (*F*(2,19) = 0.7131; *P* > 0.05) (Figure 5 and Table 3). [13] encountered a similar problem. By analysing the parameter of filtering time windows; Pascolo et al. reached a conclusion that the nonlinear characteristics from COP cannot distinguish between Parkinson's disease patients and normal controls. Of course, this method makes it more difficult to distinguish the data of normal subjects in this experiment. Since single accelerometer data are also considered as a one-dimensional time series, the same effect occurs on the LLE values from accelerometer data. All the *P* values of each body segment are greater than the significant difference level of 0.05 (Table 3).

When a subject is fighting against a sinusoidal stimulus, he/she typically changes sway speed to match the periodic perturbation, and the amplitude of high-frequency sway increases obviously (Figure 2). If human body is regarded as an inverted pendulum [33], which is controlled by a PID or PD controller, high-frequency sway may originate from the system overshoot fighting against the perturbation. According to the automatic control theory, the overshoot characteristics are determined by both amplitude of perturbation and properties of the controller. The phenomenon implies that the bigger the amplitude of perturbation is, the lager the amplitude of the high-frequency sway increases. Thus a more complex signal is generated by a superposition of perturbation and overshoot sway, and by using (3), the LLE values (for the COP or acceleration time series) show an increasing trend.

*(2) Can Individuals with Different Balance Ability Be Distinguished by the LLE?* Methods of nonlinear time series analysis are usually focused on single variable time series, but in actual problem, a given single variable time series may not be sufficient to reconstruct a complex dynamical system [17, 19]. Standing balance is the ability that enables the central nervous system (CNS) to coordinate all body segments, which consists of a multiseries structure with a motion coupling relationship. This capability cannot be completely expressed by the dynamical data of just one part of body; therefore, single time series of COP and the acceleration data only contain partial characteristics of chaotic systems.

In Tables 1 and 2, most LLE values for passive standing condition have a significant increment compared with the LLE for quiet standing condition, but the increment does not show deterministic regular. It perhaps implies a more complex motion relationship among back, hip, and knee. Assuming these irregular variations of LLE are indirectly related with the hidden dynamic properties of the human standing balance, CLLE is defined to represent the difference of LLE values for back, hip, and knee, which can compensate the defect of LLE from the one-dimensional time series.

In Figure 6(b), the boundary at f6 is significant, and CLLE is larger on the right, where it contains all the subjects in unstable group. The subjects in excellent and stable groups are on the left of the boundary, which implies that the subjects with good balance are those with smaller CLLE values. But in Figure 6(a), there are no obvious trends to express the relationship between balance ability and different subjects. The results indicate that the given metric method, based on multidimensional time series is more beneficial than the one-dimensional method, and passive standing CLLE values of most subjects show greater consistency with their balance performance. Subjects f3, f5, f6, and f8 in Figure 6(a) are close to the left part, while these same subjects are gathered close to the right part in Figure 6(b), but there is no obvious change in other subjects. This difference may demonstrate that, when individuals with poor balance suffer a sinusoidal interference, their potential dynamic characteristics become more obvious, whereas, while they are in quiet standing mode, their partly chaotic characteristics are hidden.

In Figure 7, the mean LLE_COP_ values among the groups show no significant difference (*F*(2,19) = 0.713; *P* = 0.512), while the differences of mean CLLE values among groups are significant (*F*(2,19) = 12.67; *P* = 0.000427). There is a large CLLE mean value in the unstable group (0.55), while the standard deviation is small (0.0452). Since the *P* value of CLLE values among the groups is much less than 0.05, a definitive result that the subjects in the unstable group can be effectively distinguished by passive standing CLLE values is obtained (stable versus unstable: *F*(1,13) = 13.832; *P* = 0.00293; excellent versus unstable: *F*(1,10) = 30.376; *P* = 0.0003746). But the mean CLLE values between the stable group and the excellent group show no significant difference (*F*(1,14) = 3.540; *P* = 0.08248), because the standard deviation values of both groups are large (0.1315 and 0.1237).

The traditional viewpoint is that a single variable contains overall dynamics of the system [29, 34]; therefore the dynamic process of any segment should contain whole dynamic characteristics of human structural model. However, in this research a different conclusion that the nonlinear parameters (the LLE_back_, LLE_hip_, LLE_knee_, and LLE_COP_ in the passive standing or quiet standing condition) are unable to show good classification characteristics of subjects in different groups is obtained.

One-dimensional time series (COP and the acceleration) only contain partial characteristics of chaotic systems. In fact, this single-dimension time series is more suitable to describe the dynamic characteristics of an inverted pendulum, as has been verified by simulation results based on the inverted pendulum model [33]. However, multisegment as the body is, the inherent control mechanisms of balance are related with the coordination of all body segments. Some researchers have established multisegment body models in order to study complex process. Reference [20] investigated equilibrium maintenance during standing. The body was treated as a three-joint (ankle, knee, and hip) sagittal model, and each equilibrium Eigen-movement involved different eigenvectors by independent feedback control.

In addition, CNS may take some specific control strategies to affect the behaviours. Reference [35] suggests that the body will take some specific control strategies to reduce the complexity of posture control, which may be the reason for the failure to describe the subjects' characteristics. Different selections of control strategies diversify individuals' dynamic process, so one-dimensional time series is not sufficient to characterize a whole system dynamic. In other words, it is not enough to extract balance-related characteristics from a one-dimensional time series, and balance-related features need information in more variables and more comprehensive analysis method. One could not simply suggest that the LLE values are associated with balance function, which is implicit in the individual's physical coordination. CLLE value, which is an ingenious usage of LLE from one-dimensional data, reflects the overall coordination between multisegment movements. Balance performance is consistent with passive standing CLLE, and individuals with poor balance can be distinguished by their passive standing CLLE values.

## Figures and Tables

**Table 1:** The LLE and CLLE of all subjects in quiet standing (stimulus frequency 0 Hz).

Subject	LLE_back_	LLE_hip_	LLE_knee_	LLE_COP_	CLLE	Subject	LLE_back_	LLE_hip_	LLE_knee_	LLE_COP_	CLLE
f1	0.2732	0.5066	0.4790	0.4971	0.3124	m1	0.2553	0.4237	0.4051	0.3282	0.2260
f2	0.2174	0.3764	0.4544	0.4237	0.2959	m2	0.1339	0.3227	0.2504	0.5113	0.2334
f3	0.2278	0.1263	0.2151	0.4273	0.1355	m3	0.1654	0.06458	0.2447	0.2414	0.2212
f4	0.3640	0.2462	0.2774	0.4089	0.1494	m4	0.4070	0.3934	0.4609	0.4235	0.0874
f5	0.1243	0.2066	0.1985	0.2300	0.1109	m5	0.2238	0.2821	0.3495	0.4351	0.1541
f6	0.4022	0.4485	0.3913	0.2746	0.0744	m6	0.4231	0.3992	0.3250	0.3363	0.1253
f7	0.1885	0.3252	0.3707	0.4207	0.2323	m7	0.1500	0.2818	0.2691	0.4999	0.1781
f8	0.2599	0.2422	0.3232	0.2252	0.1043	m8	0.2445	0.2595	0.5264	0.2858	0.3885
f9	0.1305	0.4150	0.2204	0.3495	0.3562	m9	0.1280	0.3823	0.3858	0.3662	0.3622
f10	0.2169	0.3252	0.2463	0.3903	0.13721	m10	0.1167	0.2128	0.4376	0.4359	0.4034

**Table 2:** The LLE and CLLE of all subjects in passive standing (stimulus frequency 0.8 Hz).

Subject	LLE_back_	LLE_hip_	LLE_knee_	LLE_COP_	CLLE	Subject	LLE_back_	LLE_hip_	LLE_knee_	LLE_COP_	CLLE
f1	0.3855	0.3838	0.2332	0.5179	0.2142	m1	0.1894	0.2301	0.1665	0.4718	0.0789
f2	0.2885	0.5878	0.5645	0.4242	0.4078	m2	0.0537	0.5449	0.3024	0.6656	0.6016
f3	0.2727	0.7342	0.4584	0.5442	0.5688	m3	0.4089	0.4656	0.4945	0.4832	0.1067
f4	0.5041	0.7327	0.6393	0.6037	0.2815	m4	0.4183	0.4181	0.6809	0.7080	0.3714
f5	0.358	0.5169	0.7770	0.4411	0.5181	m5	0.2728	0.4539	0.6235	0.7158	0.4295
f6	0.2254	0.4229	0.6253	0.6806	0.4898	m6	0.8176	0.8314	0.6437	0.2343	0.2562
f7	0.4562	0.4807	0.6409	0.7201	0.2457	m7	0.2905	0.2494	0.1796	0.4664	0.1374
f8	0.3143	0.7668	0.6428	0.8193	0.5728	m8	0.2047	0.1855	0.4014	0.5914	0.2927
f9	0.2008	0.1477	0.5819	0.4927	0.5802	m9	0.2261	0.3457	0.3571	0.6558	0.1777
f10	0.3858	0.5829	0.4403	0.3829	0.2493	m10	0.1121	0.2379	0.4534	0.6506	0.4228

**Table 3:** The average quiet standing LLE values from acceleration data (different body segment versus different group). The average LLE values from the acceleration data of body segment of excellent, stable, and unstable group are compared. The column represents the LLE values of different body segment, and the row represents different group.

Group	LLE_back_	LLE_hip_	LLE_knee_
Excellent	0.3419 ± 0.1126	0.4193 ± 0.1830	0.3894 ± 0.2220
Stable	0.3456 ± 0.2106	0.4242 ± 0.2223	0.5293 ± 0.1184
Unstable	0.2448 ± 0.1176	0.5971 ± 0.1475	0.5612 ± 0.1836
*P* value	0.9859	0.4932	0.1646
